# Predictors of early-onset post-ischemic stroke depression: a cross-sectional study

**DOI:** 10.1186/s12883-017-0980-5

**Published:** 2017-11-17

**Authors:** Guilin Meng, Xiaoye Ma, Lei Li, Yan Tan, Xiaohui Liu, Xueyuan Liu, Yanxin Zhao

**Affiliations:** Department of Neurology, Shanghai Tenth People’s Hospital, Tongji University School of Medicine, Shanghai, 200072 China

**Keywords:** Acute ischemic stroke, Inflammatory cytokine, Monoamine neurotransmitter, Nerve growth factor, Post-stroke depression

## Abstract

**Background:**

Post-stroke depression (PSD) seriously affects the rehabilitation of nerve function and quality of life. However, the pathogenesis of PSD is still not clear. This study aimed to investigate the demographic, clinical, and biochemical factors in patients with PSD.

**Methods:**

Patients with an acute ischemic stroke, who met the inclusion criteria at Shanghai Tenth People’s Hospital from April 2016 to September 2016, were recruited for this study. The stroke severity was assessed using the National Institutes of Health Stroke Scale (NIHSS), and the mental state was assessed using Mini-Mental State Examination (MMSE), Hamilton Depression Scale (HAMD), and Hamilton Anxiety Scale (HAMA) at 1 week of admission. The patients were divided into PSD and non-PSD groups. The demographic and clinical characteristics, as well as the biochemical factors, were compared between the two groups. A logistic regression analysis was performed to identify the risk factors for depression following stroke.

**Results:**

A total of 83 patients with acute ischemic stroke were recruited. Of these, 36 (43.4%) developed depression. The multivariate logistic regression analysis indicated that high NIHSS [odds ratio (OR): 1.84, 95% confidence interval (CI): 1.09–3.12, *P* = 0.023] and high HAMD scores (OR: 2.38, 95% CI: 1.61–3.50, *P* < 0.001) were independent risk predictors for PSD and so were lower dopamine level (OR: 0.64, 95% CI: 0.45–0.91, *P* = 0.014), lower 5-hydroxytryptamine level (OR: 0.99, 95% CI: 0.98–1.00, *P* = 0.046), higher tumor necrosis factor-α level (OR: 1.05, 95% CI: 1.00–1.09, *P* = 0.044), and lower nerve growth factor level (OR: 0.06, 95% CI: 0.01–0.67, *P* = 0.022).

**Conclusions:**

The identification of higher NIHSS scores, higher HAMD scores, lower dopamine level, lower 5-hydroxytryptamine level, higher tumor necrosis factor-α level, and lower nerve growth factor level might be useful for clinicians in recognizing and treating depression in patients after a stroke.

## Background

Post-stroke depression (PSD) is a common mental disease after stroke onset, mainly manifested as depression, sleep disorders, decreased interest and worthlessness, and even suicidal tendencies, accounting for one third of all patients with stroke [1]. PSD can significantly affect the recovery of neurological function in patients with stroke, significantly reduce the quality of life, and increase mortality [2].

As a result, predicting the occurrence of PSD after primary treatment is important not only for counseling the patients about the disease prognosis but also for applying additional treatment.

However, it is hard to identify the consistent risk factors from the literature. Large studies have paid much attention to the vascular factors [3, 4] and socioeconomic risk factors [5], but minimal attention has been paid to the biological factors. Intracerebral neurotransmitters have been directly related to PSD, especially monoamine neurotransmitters including noradrenaline (NE), 5-hydroxytryptamine (5-HT), and dopamine (DA), which are important neurotransmitters closely related to human mental activity, especially emotional activity [6]. Plasma concentrations of neurotransmitters seemed to be closely related to intracerebral concentrations [7], suggesting that plasma concentrations could be used to predict the cerebral concentrations using much less invasive procedures [8]. A hypothesis suggests that immune imbalance is implicated in the pathophysiology of PSD and that IL-6 and TNF-α are key cytokines [9]. Interestingly, nerve growth factor (NGF) [10] and calcitonin gene–related peptide (CGRP) [11] have been reported as relevant factors for depression. Besides, the relationship between the lesion site in the brain and PSD is also controversial [12, 13]. Therefore, a better understanding of biological factors associated with PSD is urgently required.

This retrospective study was conducted on Chinese patients to systematically investigate the correlations of depression development 1 week after ischemic stroke with the following factors: intracerebral neurotransmitters, inflammatory cytokines, NGF, CGRP, and lesion site in the brain.

## Methods

### Study design

**Fig. 1:**
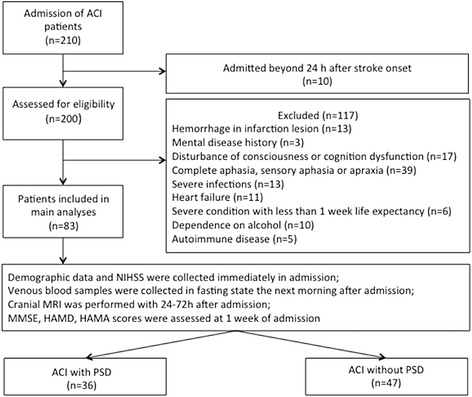
Flow chart of the study implementation. ACI, Acute cerebral infarction; NIHSS, National Institutes of Health Stroke Scale; MMSE, Mini-Mental State Examination; HAMA, Hamilton Anxiety Scale; HAMD, Hamilton Depression Scale; PSD, post-stroke depression

This study was approved by the local ethics committee of Shanghai Tenth People’s Hospital. The continuous inpatient electronic medical records at the Department of Neurology, Shanghai Tenth Hospital, were reviewed for an acute cerebral infarction (ACI) between April 2016 and September 2016. After a detailed evaluation with inclusion and exclusion criteria, 83 patients were included in this study (Fig. 1).

### Inclusion criteria

Patients who met all of the following inclusion criteria were eligible for the study:

1. Patients who fully understood the purpose of this study, expressed voluntary participation, agreed to sign informed consent, and were willing to bear the relevant risks.
2. Patients who met the criteria proposed at the Fourth Cerebrovascular Disease Conference held by the Chinese Medical Association and were diagnosed with ACI by computed tomography (CT) or magnetic resonance imaging (MRI)
3. Patients who were admitted to hospital within 24 h after stroke onset.

### Exclusion criteria

1. Presence of intracranial hemorrhage or subdural hematoma evidenced by cranial CT scan
2. Presence of depression-positive mental disorder within the previous 6 months
3. Presence of disturbance of consciousness or serious cognitive dysfunction
4. Presence of complete aphasia, sensory aphasia, or apraxia
5. Presence of severe infectious diseases such as respiratory system infections, urinary system infections, and gastrointestinal infections; severe heart failure; liver and kidney disease; blood disorder; immune disease; thyroid disease; epilepsy; or cancer
6. Severe condition with a life expectancy of less than 1 week
7. Pregnant or lactating women
8. Dependence on alcohol, tobacco, or other substances
9. Presence of autoimmune diseases or mental retardation.

At admission, demographic data and history of conventional vascular risk factors were recorded.

### Testing indexes of plasma concentrations

Venous blood samples were collected the next morning after admission for basic biochemical tests, fasting and postprandial blood glucose, glycosylated hemoglobin (HbAlc), low-density lipoprotein (LDL), high-density lipoprotein (HDL), and thyroid function. Samples were marked with a unique study number only. Some of the fasting blood was centrifuged, and the serum was stored at −80°C. The concentrations of norepinephrine (NE), 5-HT, dopamine (DA), CGRP, and NGF were detected using enzyme-linked immunosorbent assay.

### Detailed assessment and grouping

Stroke severity was assessed using the National Institutes of Health Stroke Scale (NIHSS) [14] at the time of admission. The mental state was assessed using Mini-Mental State Examination (MMSE), Hamilton Depression Scale (HAMD), and Hamilton Anxiety Scale (HAMA) by trained neurologists at 1 week of admission.

CT brain scans were obtained routinely in the emergency room, and it was also possible that the patient was sent to the inpatient Neurology department directly before the cranial CT if the symptoms and signs were quite typical, thus in this situation, cranial CT was performed in Neurology department immediately after admission. Cranial MRI was performed within 24–72 h after admission to assess the site of the brain infarct.

Patients with HAMD scores greater than or equal to 8 were included in the PSD group [15]. Patients with HAMD scores less than 8 were enrolled in the non-PSD group. Finally, the 2 groups included 36 and 47 patients, respectively.

### Statistical analysis

The Kolmogorov–Smirnov test was used to determine whether the metrological data followed the normal distribution. Continuous variables, which followed a normal distribution, were expressed as mean ± standard deviation (x ± s). Patients with PSD and without PSD were compared using the independent two-sample *t* test or Mann–Whitney *U* test. The chi-square/Fisher’s exact test was used for categorical variables. Variables having a *P* value less than 0.1 in the univariate analysis were selected and evaluated using multivariate logistic regression models with the conditional forward selection method to minimize confounding and examine their independent contributions of what we adjusted for. All statistical assessments were two tailed, and a *P* value less than 0.05 was considered statistically significant. Statistical analyses were performed using SPSS 22.0 statistical software (SPSS Inc., IL, USA).

## Results

### General patient characteristics

A total of 210 patients presented to the Neurology department at the Shanghai Tenth Hospital for ACI between April 2016 and September 2016. Eighty-three of them were found to fulfill the criteria for further analysis.

The average age was 69.4 years (range 50–86 years); 51.8% were men (Table 1). Fifty-nine patients (71.1%) had a history of hypertension, 38 (45.8%) had diabetes mellitus, and 13 (15.7%) had atrial fibrillation.

**Table 1:** Baseline characteristics of patients with ACI and with and without PSD

Characteristics	All Patients (*n* = 83)	PSD (*n* = 36)	Non-PSD (*n* = 47)	*P* value
Age, y, mean (SD)	69.0(9.9)	67.9(9.1)	69.8(10.4)	0.397
Sex				
Male	43(51.8)	22(61.1)	21(44.7)	0.138
Female	40(48.2)	14(38.9)	26(55.3)	
Medical history				
Hypertension	59(71.1)	24(66.7)	35(74.5)	0.437
Diabetes mellitus	38(45.8)	16(44.4)	22(46.8)	0.830
Atrial fibrillation	13(15.7)	4(11.1)	9(19.2)	0.318
Lesion location				
Frontal	24(28.9)	15(41.7)	9(19.2)	0.025^*^
Temporal	8(9.6)	4(11.1)	4(8.5)	0.982
Parietal	13(15.7)	6(16.7)	7(14.9)	0.049^*^
Occipital	7(8.4)	3(8.3)	4(8.5)	1.000
Basal ganglia	42(50.6)	19(52.8)	23(48.9)	0.729
Cerebellum	3(3.6)	1(2.8)	2(4.3)	1.000
Brain stem	12(14.2)	4(11.1)	8(17.0)	0.448
Corona radiata	34(41.0)	16(44.4)	18(38.3)	0.573
Neurophysiological test scores, median (IQR)				
NHISS	1(1–2)	3(1–6)	1(0–2)	< 0.001^#^
HAMD	2(0–6)	9.4(8.5–10.8)	0(0–1)	< 0.001^#^
HAMA	2(0–7)	6.5(5–9)	0(0–1)	< 0.001^#^
MMSE	27(25–30)	25(22–27)	29(26–30)	< 0.001^#^

*P* values comparing persons with and without PSD. ^*^ *P* < 0.05 vs non-PSD group; ^#^ *P* < 0.001 vs non-PSD group

Numbers (%) are provided unless otherwise specified

*HAMA* Hamilton Anxiety Scale, *HAMD* Hamilton Depression Scale *IQR* interquartile range, *MMSE* Mini-Mental State Examination, *NIHSS* National Institutes of Health Stroke Scale, *PSD* post-stroke depression, *SD* standard deviation

Thirty-six (43.4%) of the 83 enrolled patients with ACI were diagnosed as having PSD during their hospitalization. In general, patients with PSD were more likely to present with higher NIHSS scores (median, 3 vs 1), higher HAMD scores (median, 8 vs 0), higher HAMA scores (median, 6.5 vs 0), and lower MMSE scores (median, 25 vs 29) than did patients without PSD (Table 1), indicating that the neurological deficits were more serious in the PSD group. PSD was more likely to occur in patients with frontal lesions (41.7% vs 19.15%) and parietal lesions (16.7% vs 14.9%).

### Tests for biochemical indicators

Several laboratory tests were conducted to examine the differences in biochemical indicators between the two groups. As a result, patients with PSD were more likely to present with lower TT3 levels (average 1.1 vs 1.3 μg/L), but no significant difference in thyroid-stimulating hormone (TSH) levels was reported (Table 2).

**Table 2:** Biochemical indicators of patients with ACI and with and without PSD

Variables, mean(SD)	PSD (*n* = 36)	Non-PSD (*n* = 47)	*P* value
TT3, μg/L	1.1(0.2)	1.3(0.2)	< 0.001^#^
TT4, μg/L	79.8(10.6)	81.1(11.0)	0.587
TSH, uIU/mL	1.6(0.9)	1.5(0.7)	0.629
LDL, mmol/L	2.5(0.9)	2.6(0.9)	0.622
HDL, mmol/L	1.2(0.3)	1.2(0.4)	0.549
FBG, mmol/L	6.8(2.2)	6.3(2.0)	0.338
2hPBG, mmol/L	10.2(4.6)	9.6(4.5)	0.548
HbAlc, %	6.1(1.5)	6.2(1.8)	0.793
NE, ng/L	931.8(172.9)	1237.8(296.5)	< 0.001^#^
DA, ng/L	46.7(10.2)	64.7(8.9)	< 0.001^#^
5-HT, ng/L	821.7(228.4)	1065.9(144.3)	< 0.001^#^
IL-6, ng/L	4.6(1.0)	3.4(0.9)	< 0.001^#^
TNF-α, ng/L	224.4(44.1)	164.9(43.7)	< 0.001^#^
NGF, ng/L	6.5(1.5)	8.1(1.6)	< 0.001^#^
CGRP, pg/ml	41.9(6.1)	38.3(13.8)	0.112

*P* values compare persons with and without PSD. ^#^ *P* < 0.001 vs non-PSD group

Data are displayed as mean (standard deviation)

*CGRP* calcitonin gene-related peptide, *DA* dopamine, *FBG* fasting blood glucose, *5-HT* 5-hydroxytryptamine, *HbA1c* glycosylated hemoglobin, type A1c, *HDL* high-density lipoprotein, *IL-6* interleukin 6, *LDL* low-density lipoprotein, *NE* norepinephrine, *NGF* nerve growth factor, *2hPBG* 2-h postprandial blood glucose, *PSD* post-stroke depression, *SD* standard deviation, *TNF-α* tumor necrosis factor-α, *TSH* thyroid-stimulating hormone, *TT3* total triiodothyronine, *TT4* total thyroxine

This study also found that the monoamine neurotransmitters were significantly lower and the levels of IL-6 and TNF-α were significantly higher in the PSD group than in the non-PSD group (Table 2). Moreover, patients with PSD were more likely to present with lower NGF levels (average 6.5 vs 8.1 ng/L); no significant difference in CGRP levels was found (Table 2).

### Logistic regression analysis

Univariate logistic regression identified the following demographic and clinical characteristics associated with PSD: frontal lesions [odds ratio (OR) 3.02; *P* = 0.028], NIHSS scores (OR 2.08 per 1-point increase in NIHSS scores; *P* < 0.001), HAMD scores (OR 2.63 per 1-point increase in HAMD scores; *P* < 0.001), HAMA scores (OR 2.10 per 1-point increase in HAMA scores; *P* < 0.001), and MMSE scores (OR 0.77 per 1-point decrease in MMSE scores; *P* = 0.001)]. In multivariable logistic regression, only NIHSS scores (OR 1.84 per 1-point increase in NIHSS scores; *P* = 0.023) and HAMD scores (OR 2.38 per 1-point increase in HAMD scores; *P* < 0.001) were independent demographic and clinical predictors of PSD (*P* < 0.05) (Table 3).

**Table 3:** Univariate and multivariable analyses of demographic and clinical predictors of PSD in patients with ACI

	Univariate		Multivariate	
	OR (95% CI)	*P* value	OR (95% CI)	*P* value
Age, yrs	0.98(0.94,1.03)	0.392		
Female vs. Male	1.95(0.81,4.71)	0.140		
Hypertension				
Yes vs. no	0.69(0.26,1.78)	0.438		
Diabetes mellitus				
Yes vs. no	0.91(0.38,2.17)	0.830		
Atrial fibrillation				
Yes vs. no	0.53(0.15,1.88)	0.323		
Lesion location				
Frontal				
Yes vs. no	3.02(1.13,8.06)	0.028^*^		
Temporal				
Yes vs. no	1.34(0.31,5.78)	0.692		
Parietal				
Yes vs. no	1.14(0.35,3.75)	0.826		
Occipital				
Yes vs. no	0.98(0.21,4.67)	0.977		
Basal ganglia				
Yes vs. no	1.17(0.49,2.78)	0.729		
Cerebellum				
Yes vs. no	0.64(0.06,7.38)	0.723		
Brain stem				
Yes vs. no	0.61(0.17,2.21)	0.451		
Corona radiata				
Yes vs. no	1.29(0.53,3.11)	0.573		
Neurophysiological test scores				
NIHSS	2.08(1.42,3.06)	< 0.001^#^	1.84(1.09,3.12)	0.023^*^
HAMD	2.63(1.74,3.96)	< 0.001^#^	2.38(1.61,3.50)	< 0.001^#^
HAMA	2.10(1.56,2.83)	< 0.001^#^		
MMSE	0.77(0.65,0.90)	0.001^**^		

*CI* confidence interval, *HAMA* Hamilton Anxiety Sale, *HAMD* Hamilton Depression Scale, *MMSE* Mini-Mental State Examination, *NIHSS* National Institutes of Health Stroke Scale, *OR* odds ratio

^*^ *P* < 0.05; ^#^ *P* < 0.001

Univariate logistic regression identified the following biochemical indicators associated with PSD: TT3 (OR 0.01; *P* < 0.001), NE (OR 0.99; *P* < 0.001), DA (OR 0.85; *P* < 0.001), 5-HT (OR 0.99; *P* < 0.001), IL-6 (OR 3.23; *P* < 0.001), TNF-α (OR 1.03; *P* < 0.001), and NGF (OR 0.55; *P* < 0.001). In multivariable logistic regression, only DA (OR 0.64; *P* = 0.014), 5-HT (OR 0.99; *P* = 0.046), TNF-α (OR 1.05; *P* = 0.044), and NGF (OR 0.06; *P* = 0.022) were independent biochemical predictors of PSD (*P* < 0.05) (Table 4).

**Table 4:** Univariate and Multivariable Analysis for Biochemical Predictors of PSD in Patients With ACI

	Univariate		Multivariate	
	OR (95% CI)	*P* value	OR (95% CI)	*P* value
TT3, μg/L	0.00(0.000,0.04)	< 0.001^#^		
TT4, μg/L	0.99(0.95,1.03)	0.582		
TSH, uIU/mL	1.15(0.66,2.03)	0.625		
LDL, mmol/L	0.89(0.56,1.41)	0.618		
HDL, mmol/L	0.66(0.17,2.56)	0.438		
FBG, mmol/L	1.11(0.90,1.36)	0.337		
2hPBG, mmol/L	1.03(0.94,1.13)	0.543		
HbA1c, %	0.97(0.74,1.26)	0.790		
NE, ng/L	0.99(0.99,0.99)	< 0.001^#^		
DA, ng/L	0.85(0.79,0.90)	< 0.001^#^	0.64(0.45,0.91)	0.014^*^
5-HT, ng/L	0.99(0.99,1.00)	< 0.001^#^	0.99(0.98,1.00)	0.046^*^
IL-6, ng/L	3.23(1.90,5.48)	< 0.001^#^		
TNF-α, ng/L	1.03(1.02,1.04)	< 0.001^#^	1.05(1.00,1.09)	0.044^*^
NGF, ng/L	0.55(0.41,0.76)	< 0.000^#^	0.06(0.01,0.67)	0.022^*^
CGRP, pg/ml	1.03(0.99,1.07)	0.146		

*CI* confidence interval, *CGRP* calcitonin gene-related peptide, *DA* dopamine, *FBG* fasting blood glucose, *5-HT* 5-hydroxytryptamine, *HbA1c* glycosylated hemoglobin, type A1c, *HDL* high-density lipoprotein, *IL-6* interleukin 6, *LDL* low-density lipoprotein, *NE* norepinephrine, *NGF* nerve growth factor, *OR* odds ratio, *2hPBG* 2-h postprandial blood glucose, *PSD* post-stroke depression, *SD* standard deviation, *TNF-α* tumor necrosis factor-α, *TSH* thyroid-stimulating hormone, *TT3* total triiodothyronine, *TT4* total thyroxine

^*^ *P* < 0.05; ^#^ *P* < 0.001

The multivariable logistic regression equation was logit(P) = 1/2(− 6.36 + 0.61 × NIHSS + 0.87 × HAMD + 46.25–0.45 × DA – 0.01 × 5-HT + 0.04 × TNF-α – 2.8 × NGF).

## Discussion

This study investigated the risk factors associated with PSD, such as demographic factors, clinical characteristics, and biochemical factors. NIHSS scores, MMSE scores, HAMA scores, HAMD scores, monoamine neurotransmitters, inflammatory cytokines, NGF, and the lesion site in the brain were found to be related to PSD, whereas CGRP was not related to PSD. Importantly, the study found that NIHSS and HAMD scores were demographic and clinical characteristics independently associated with PSD. Moreover, DA, 5-HT, TNF-α, and NGF levels were biochemical indicators independently associated with PSD.

As shown in Tables 1 and 3, significant differences were found in NIHSS, HAMD, HAMA, and MMSE scores between the two groups, indicating that neurological deficits were more serious in the PSD group. Other studies also showed the same results [16, 17]. Further, a significant difference was found in frontal and parietal lesions between the two groups. Likewise, a study showed the involvement of subcutaneous pathway in the frontal lobe, especially the caudate nucleus, globus pallidus, internal capsule knee, and left superior hemisphere [12]; however, other studies showed no significant correlation between the lesion site and PSD [13, 18]. Unlike the present study, Vahid-Ansari et al. developed a preclinical model of PSD in mice by inducing a unilateral ischemic lesion in the medial prefrontal cortex after stroke [19]. The results regarding the value of lesion site in predicting disease outcome are still controversial. Therefore, retrospective trials with larger series of patients with PSD are warranted to demonstrate the value of various lesion sites.

Monoamine neurotransmitters include mainly noradrenaline (NE), 5-HT, and DA. The somata of these neurons are located in the brainstem, and the axons reach the frontal cortex through the thalamus and basal ganglia. If any of the aforementioned locations are damaged, the levels of monoamine neurotransmitters decrease, resulting in depression [20]. Likewise, as shown in Table 2, it was found that NE, 5-HT, and DA levels were significantly lower in the PSD group than in the non-PSD group, supporting the hypotheses of monoamine neurotransmitters. Similarly, reduced DA concentrations in ischemic striatum have been demonstrated in a mouse model of chronic PSD [21]. In addition to monoamine neurotransmitters, a low plasma glutamate has also been reported to be associated with early-onset PSD recently [22].

This study found the levels of IL-6 and TNF-α significantly higher in the PSD group than in the non-PSD group. Inflammatory cytokines are implicated in the pathogenesis of PSD. Spalletta et al. [23] believed that increased inflammatory cytokines after stroke induced damage in the marginal zone by activating indoleamine-2,3-dioxygenase, leading to 5-HT depletion in the secondary edge system. Besides, inflammatory cytokines can also affect the protective cytokines and some neurotransmitters in the brain, thus indirectly promoting the occurrence of PSD. Moreover, some studies have shown the overexpression of inflammatory cytokines in patients with cerebral ischemic stroke [24, 25]. Similarly, IL-6, IL-10, TNF-α, and interferon-γ levels increased to different degrees in patients with PSD, corresponding well with the result shown in the patients with PSD in the present study [9, 26]. Interestingly, increased serum IL-18 levels were also suggested as a biomarker for PSD [27].

NGF, a secretory protein first found in neurotrophic factors, inhibits apoptosis and promotes survival, growth, and differentiation of neurons [28]. Some studies have suggested increased NGF expression in cerebral ischemia [29, 30]. This study found the serum NGF levels to be significantly lower in the PSD group than in the non-PSD group. Likewise, several studies have found the NGF levels to be significantly lower in the severe depression group than in the normal control group [31, 32]. Other studies showed that elevated serum NGF levels could significantly ameliorate the depression symptoms and improve the quality of life [33, 34]. Unlike the present study, several studies suggested no correlation between depression and NGF [35, 36]. The differences in patient groups and experimental design might account for the difference in results.

Further, CGRP, an active peptide of 37 amino acids widely distributed in the nervous and cardiovascular system, has potent vasodilator and neuroprotective effects [37]. The CGRP synthesis was known to increase when nerve damage or inflammatory responses occurred [38]. The present study found that CGRP levels were higher in the PSD group than in the non-PSD group, but with no statistically significant difference. Unlike the present study, Shao et al. showed that CGRP immunoreactivity (CGRP-ir) concentration in the cerebrospinal fluid and hippocampus increased in rats with PSD and the administration of CGRP into the ischemic rats increased depression-like behaviors in a dose-dependent manner [11]. A study also found that CGRP antagonists could significantly ameliorate the depression symptoms [39]. The difference in sample size might account for the diversity in results.

Only 83 patients were examined in the present study and the stroke severity of sample was not high. Hence, the results must be confirmed by conducting large-sample studies. The reason for this minimally affected sample was the strict inclusion and exclusion criteria. It is common that patients with higher NIHSS always have aphasia with different degrees of severity, and old-age patients have multiple-system and multiple-organ disorders, and cognition dysfunction, which were all part of the exclusion criteria. However, the preliminary risk factor model provided more confidence to take action for individuals with higher stroke severities. Besides, a potential limitation of this study was the inherent differences between the participants in this clinical trial and the general population of stroke survivors. Another limitation was the absence of medical assessment record of the previous mental states before the stroke onset because the depression history was sometimes not objective for depressive patients without illness perception/cognition. Furthermore, the ideal time for testing the levels of plasma parameters in patients with acute stroke still needs further longitudinal studies. Nevertheless, the results were noteworthy because this novel study systematically investigated the correlations of depression after ischemic stroke with the following factors: neurotransmitters, inflammatory cytokines, NGF, CGRP, and lesion site in the brain.

Overall, it is speculated that the inflammatory response can aggravate the injury in ischemic regions and, meanwhile, lead to 5-HT depletion, increase in DA level, and inhibition of NGF expression, thereby promoting the development of PSD. However, the relationship between inflammatory response with PSD and ideal optimum plasma biomarkers still needs further investigation. The findings of this study might be helpful in preventing PSD and ensuring the adequacy of treatment. All stroke survivors should be screened early for depression. It is critical that patients with PSD are provided with appropriate treatment. Also, larger studies with longer follow-up should be conducted in the future.

## Conclusions

The risk factors for PSD were identified as higher NIHSS scores, higher HAMD scores, lower DA level, lower 5-HT level, higher tumor necrosis factor-α level, and lower NGF level. These results might be useful for clinicians in recognizing and treating depression in patients after a stroke.

## Abbreviations

2hPBG: 2-h postprandial blood glucose
5-HT: 5-hydroxytryptamine
ACI: Acute cerebral infarction
CGRP: Calcitonin gene–related peptide
CGRP-ir: CGRP immunoreactivity
CI: Confidence interval
CT: Computed tomography
DA: Dopamine
FBG: Fasting blood glucose
HAMA: Hamilton anxiety scale
HAMD: Hamilton depression scale
HbAlc: Glycosylated hemoglobin
HDL: High-density lipoprotein
IL-6: Interleukin 6
IQR: Interquartile range
LDL: Low-density lipoprotein
MMSE: Mini-mental state examination
MRI: Magnetic resonance imaging
NE: Noradrenaline
NGF: Nerve growth factor
NIHSS: National institutes of health stroke scale
OR: Odds ratio
PSD: Post-stroke depression
SD: Standard deviation
TNF-α: Tumor necrosis factor-α
TSH: Thyroid-stimulating hormone
TT3: Total triiodothyronine
TT4: Total thyroxine
